# Physical Activity Improves Mental Health in Children and Adolescents Irrespective of the Diagnosis of Attention Deficit Hyperactivity Disorder (ADHD)—A Multi-Wave Analysis Using Data from the KiGGS Study

**DOI:** 10.3390/ijerph18052207

**Published:** 2021-02-24

**Authors:** Parisa Ganjeh, Thomas Meyer, York Hagmayer, Ronny Kuhnert, Ulrike Ravens-Sieberer, Nicole von Steinbuechel, Aribert Rothenberger, Andreas Becker

**Affiliations:** 1 Department of Child and Adolescent Psychiatry and Psychotherapy, University Medical Center Göttingen, 37075 Göttingen, Germany; arothen@gwdg.de (A.R.); abecker4@gwdg.de (A.B.); 2 Department of Psychosomatic Medicine and Psychotherapy, University Medical Center Göttingen, 37073 Göttingen, Germany; thomas.meyer@med.uni-goettingen.de; 3 German Centre for Cardiovascular Research (DZHK), Partner Site Göttingen, 37073 Göttingen, Germany; 4 Department of Cognitive Science and Decision Psychology, Georg Elias Müller Institute of Psychology, University of Göttingen, 37073 Göttingen, Germany; york.hagmayer@bio.uni-goettingen.de; 5 Department of Epidemiology and Health Monitoring, Robert Koch Institute, 13353 Berlin, Germany; kuhnertr@rki.de; 6 Department of Child and Adolescent Psychiatry, Psychotherapy and Psychosomatics, University Medical Center Hamburg-Eppendorf, 20246 Hamburg, Germany; ravens-sieberer@uke.uni-hamburg.de; 7 Institute of Medical Psychology and Medical Sociology, University Medical Center Göttingen, 37073 Göttingen, Germany; nvsteinbuechel@med.uni-goettingen.de

**Keywords:** ADHD, adolescents, children, KiGGS, long-term effects, mental health, physical activity, Strengths and Difficulties Questionnaire

## Abstract

Physical activity (PA) may have positive effects on mental health in children and adolescents. This post hoc study aimed to further investigate the relationship between different frequency levels of PA and general mental health as well as specific hyperactivity/inattention symptoms in children and adolescents. Methods: The analyses were based on data drawn from the German Health Interview and Examination Survey for Children and Adolescents (KiGGS) study, a regularly conducted large-scale, epidemiological investigation of somatic and mental health of children and adolescents in Germany. Parents were asked about their children’s attention deficit hyperactivity disorder (ADHD) records and answered questionnaires concerning any mental health problem behavior of the children and adolescents using the Strengths and Difficulties Questionnaire (SDQ). The overall problem score as well as the hyperactivity/inattention symptoms subscale (SDQ-H/I) were entered as outcomes in a regression model controlling for parental socio-economic status and participants’ sex, age, and body mass index (BMI). Cross-sectional analyses were conducted at three time points of the KiGGS study (baseline, wave 1, and wave 2) using general linear models (GLM). This was performed for different age groups (4–5, 6–9, 10–17 years). Results: Significant negative relationships were found between PA and general mental health problems. For the relationship between PA and SDQ-H/I, different patterns emerged at the three time points. There was no interaction between PA frequency levels and diagnosis of ADHD (ADHD vs. non-ADHD controls) regarding the SDQ total score. Conclusion: This study underlines the importance of a high frequency level of PA for a good mental health status among children and adolescents, irrespective of the diagnosis of ADHD.

## 1. Introduction

For adults, it is already known that, in general, physical activity (PA) is effective for the prevention and treatment of somatic diseases including heart diseases, cancers, diabetes mellitus, stroke, and overweight [1,2]. In contrast, physical inactivity may have negative consequences for individuals, family, and the community, directly and indirectly increasing costs to the healthcare system [1]. Several reports have shown that PA not only improves physical health but also may have a positive impact on mental health problems, well-being, and quality of life [3,4,5,6,7,8,9]. Although the number of studies investigating PA and mental illness in adults is increasing [10,11,12,13,14,15,16,17], results pertaining to this relationship in children and adolescents are mixed and even scarce. Hence, more studies are needed in order to obtain a clearer picture before firm conclusions can be drawn, specifically those addressing PA for prevention and treatment of developmental psychopathology, either in general or for a specific disorder. In non-adult populations, PA may lead to improvement of emotional mental health and social support, and also may raise self-esteem [18,19,20,21,22].

There are several empirical studies suggesting positive effects of PA on mental health among children and adolescents [23,24,25,26,27,28,29,30,31,32], however, contradictory results have also been published [33,34]. In a cross-sectional study, O’Brien et al. investigated the relationship between PA and the risk of psychological problems among early school-aged children and found no significant association [35]. Other studies, conducted among young children, found limited evidence for an inverse negative correlation between sports or PA and emotional symptoms/conduct problems, hyperactivity/inattention, and behavioral problems [36,37]. In their review and meta-analysis of the effect of PA interventions on mental health outcomes in preschoolers (2–5 years of age), elementary-school children (6–11 years of age), and adolescents (12–18 years of age), Rodriguez-Ayllon et al., demonstrated a small but significantly beneficial effect of PA on mental health in 6- to 18-year-old children and adolescents (Cohen’s d = 0.173) [38]. However, when they performed separate analyses for children and adolescents, the results were significant for the latter but not for the former. Moreover, it is not clear whether PA is more relevant for general or rather only for certain specific psychopathologies. Therefore, further research on different age groups and mental health problems seems to be essential before firm conclusions for practical interventions may be drawn.

With regard to psychopathological specificity and clear relatedness of motoric restlessness to PA, we referred to one of the most common and practically important externalizing psychiatric disorders in childhood and adolescence, namely, attention deficit hyperactivity disorder (ADHD), which is characterized by age-inappropriate degrees of inattention, impulsivity, and hyperactivity, frequently leading to long-term academic, social, and mental health problems [39,40]. The usual first line treatment for ADHD differs depending on age. For preschool children, behavior therapy is the most commonly recommended treatment. For elementary school children and adolescents, medications are the major line of treatment [41]. However, there can be negative side effects of psychopharmacological treatment [42,43]. Moreover, about one-third of children may not experience a sufficient reduction in ADHD symptoms after stimulant medication and behavioral treatment [44]. Possibly, PA might play a prominent role as an alternative and/or supplementary treatment and protective factor for children with ADHD [45].

A number of studies, reviews, and meta-analyses have already documented some benefits of PA for ADHD-diagnosed children, including reductions in ADHD symptoms, improved academic achievement, as well as socio-emotional and cognitive performance [46,47,48,49,50,51,52,53,54,55,56,57,58,59]. For example, an intervention study reported major reduction in ADHD symptoms among children during PA sessions, observed by teachers in school. Moreover, these teachers reported a general increase in involvement in the classroom educational activities [57]. A recent meta-analysis provides evidence that physical exercise may lead to a significant reduction in anxiety and depression symptoms, aggressive behavior, and social problems in ADHD children [58].

When studying health behaviors and ADHD in children aged 11 to 17 years van Egmond-Fröhlich and colleagues observed a weak but significant positive relationship between the child’s self-report of medium-high intensity PA and the parent-rated hyperactivity/inattention score [60]. In addition, several meta-analyses reported that the positive results of many studies have to be interpreted with caution because of various factors, including low effect sizes, large differences in study designs and group compositions (specifically, no comparison of different age groups), small sample sizes, small number of studies, no randomization or blinding method, lack of sufficient control conditions, no healthy control group, risk of bias, and the heterogeneity of their outcome measures [45,48,61]. In addition, the present evidence is insufficient to provide a clear positive statement for different age groups during childhood and adolescence and recommendation for PA as part of a treatment program in children and adolescents with mental health problems [45,48,52,58,61].

The present study addressed some of the limitations of previous work and aimed to broaden and deepen the existing knowledge. To this end, we used epidemiological behavioral data (including PA) of a large sample size with different age groups of children and adolescents at three different time points and measured child and adolescent mental health problem behavior in general and specific aspects in order to test whether PA may have a differentiating effect. Studying groups of children at different periods of their development can provide us with more reliable results about the effect of PA on mental health in children and adolescents. Hence, if the same results were repeated at different time points, we could infer that the effect of PA on mental health of children and adolescents works irrespective of the time period and the kind of psychopathology in question, supporting the reliability of the observed results.

The aims of this study were threefold: (1) to examine the relationship between PA and general mental health problems in different age groups in children and adolescents, (2) to investigate the association between PA and ADHD symptoms during development from childhood to early adulthood, and (3) to assess the role of ADHD diagnosis (ADHD as compared to non-ADHD controls) as a possible moderator of the association between different levels of PA and mental health problems in a wide age range of German children and adolescents.

## 2. Materials and Methods

### 2.1. Participants and Procedures

Since 2003, the Robert Koch Institute (RKI) has followed the health of children and adolescents in Germany, investigating representative samples of children into late adolescence/young adulthood in the regularly conducted cross-sectional part of the German Health Interview and Examination Survey for children and adolescents (KiGGS). Aims, background, and design of the KiGGS study are described in detail elsewhere [62,63,64]. In brief, 17,640 girls and boys aged from aged from 0 to 17 years and their parents participated in the KiGGS baseline survey from 2003 to 2006. Participants were randomly selected at 167 study sites using a stratified sampling method. The response rate was 66.6%. Six years later, KiGGS wave 1 (2009–2012) was conducted via telephone interviews. The sample of KiGGS wave 1 consisted of a new cross-sectional sample of 0- to 6-year-old children randomly drawn from the population registers of the original 167 study sites. In addition, the earlier participants of the KiGGS baseline survey were again invited to the survey, who were then 6 to 24 years old and continued as a closed cohort. Overall, 12,368 children and adolescents (6093 girls, 6275 boys) participated, with an age range from 0 to 17 years, including 4455 first-timers (response rate 38.8%) and 7913 re-invited participants (response rate 72.9%) [65]. Overall, 15,023 children and adolescents aged from 0 to 17 years participated in KiGGS wave 2 (2014–2017), which included a physical examination. There were 10,853 girls and boys from the baseline group who were part of the longitudinal sample of the second wave (with a response rate of 61.5%) [66].

### 2.2. Selection of Groups

Three different age groups were selected (preschool children, elementary school children, and adolescents) on the basis of different psychosocial developmental aspects and the challenges that these age groups face. By keeping the same participants over a time course of about 10 years, analysis of these longitudinal data gives us a chance to compare the results between the separate waves with no need to pair the participants or to control the disturbing variables. In a first step, the longitudinal data for the age range from 4 to 17 years were used to run cross-sectional analyses at the three measurement points. Hence, we had to leave out the data of children older than 17 years at a certain measurement point. Therefore, we could analyze data of three age groups at baseline (4–5, 6–9, 10–17 years), two age groups at wave 1 (6–9, 10–17 years), and only one group at wave 2 (10–17 years). Because ADHD can be validly diagnosed only from the age of 4, our sample selection was taken from that age.

### 2.3. Measurements

Physical activity: Data for PA were obtained from parents’ answers to one question given at baseline (“In how much sport and physical activity does your child take part?”). There were three response categories, namely 1 = low, 2 = medium, and 3 = high. Physical activity was measured through a parent-reported question according to WHO recommendation for children and adolescents at waves 1 and 2. The question was “On how many days is your child at least 60 min physically active during a normal week?”. The response range was from 0 = never to 7 = 7 days a week. The response range of the question at waves 1 and 2 was categorized according to the baseline in order to have a better comparison between time points (never to 2 days per week = low, 3 to 5 days per week = medium, and 6 to 7 days per week = high). PA was used as a categorical variable with three frequency levels (low, medium, and high) in this study.

Mental health problems and ADHD symptoms: In the KiGGS study, mental health was evaluated by parent- and self-report using the Strengths and Difficulties Questionnaire (SDQ) [67]. The SDQ includes 25 items with statements that are rated from 0 = not true to 2 = certainly true. The SDQ has five subscales, namely emotional problems, conduct problems, hyperactivity/inattention, peer problems, and prosocial behavior. The score of each subscale ranges between 0 and 10. The total difficulties score is obtained as the sum of the four subscales (including all, except for prosocial behavior) and ranges between 0 and 40. A higher score indicates more difficulties. In the current study, the parent-report of total difficulties and the hyperactivity/inattention subscale (SDQ-H/I) were used as indicator for general mental health problems and symptoms of ADHD, respectively. Psychometric properties of the questionnaire were investigated in normal [68] and clinical [69] samples of German children and adolescents. The scores are reliable and highly correlated with the Child Behavior Checklist (CBCL). The questionnaire is valid to distinguish psychiatric patients and different categories of disorders in the clinical sample [68,69]. In the present study, Cronbach’s alphas for total score of SDQ and SDQ-H/I subscale were 0.80 and 0.76, respectively.

In the KiGGS study, parents were asked “Has your child ever been diagnosed with an attention deficit/hyperactivity disorder by physicians or psychologists?”. This item was used for grouping children and adolescents as diagnosed with ADHD versus a control group without diagnosis. Parental socio-economic status [70] and participants’ sex, age, and body mass index (BMI) were used as covariates in the analyses. The correlation between covariates and dependent variables was estimated at each age category, and if there was a significant correlation among them, the covariate was used in the analysis.

### 2.4. Analyses

This cross-sectional, post hoc analysis was conducted at each time point separately according to available data for PA, SDQ, and the diagnosis of ADHD. Three kinds of analyses were run: (1) comparison of the SDQ score between frequency levels of PA, (2) comparison of the SDQ-H/I score between frequency levels of PA, and (3) interactions between PA and diagnosis of ADHD (ADHD vs. control) taking the SDQ score into account. Predictors were categorical (three levels of PA in the association and interaction analyses and two groups of ADHD diagnosis in the interaction analysis) and outcomes were continuous (total SDQ and SDQ-H/I scores). Therefore, a general linear model (GLM) was used to analyze the data. Before running the analyses, we checked assumptions of GLM (homoscedasticity and normal distribution). If the assumptions were not met, outcomes were transformed by using the square-root method. Moreover, the normality of the distribution of the residual was tested. These checks had an effect on the making of the decision to transform the data. If the standardized values of residuals were larger than ±3, the data points were removed from the analysis (the deleted data were fewer than 1%). If, after transforming, the homogeneity of variance was still not met, a general least square model (GLS) taking the heterogeneity into account was used. After fitting a model, differences in outcomes between the three frequency levels of PA were analyzed by between-group contrasts. A negative estimate means that the total SDQ score or SDQ-H/I score was higher at a lower level of PA. To analyze for an interaction between frequency levels of PA and the parent-reported diagnosis of ADHD (ADHD vs. controls) with respect to the total SDQ score, we compared a model including the interaction term to a model not including the interaction term. Because there was a large difference between the number of children diagnosed with ADHD and controls, a propensity score matching was used in order to obtain a reliable result in the interaction analysis. Matching for ADHD diagnosis groups was conducted according to a PA variable. All analyses were conducted using R including the packages haven, car, MatchIt, psych, and nlme [71], and IBM SPSS Statistics for Windows, Version 26.0 (International Business Machines Corporation, New York, NY, USA).

## 3. Results

### 3.1. Descriptive Statistics

According to a meta-analysis that found different results for children and adolescents in separate analyses [38], we chose different age groups in order to better compare the results. The total numbers of children and adolescents at baseline, wave 1, and wave 2 were 13,901, 8501, and 4598, respectively. The descriptive statistics regarding the analysis of the different age groups for each of the three time points is reported in Table 1, Table 2 and Table 3.

Ultimately the SDQ total score had to be transformed using the square-root technique (SQRT), whereas the SDQ-H/I score did not have to be transformed. Only the SDQ-H/I score in the 6- to 9-year-old age group at wave 1 was transformed by employing the square-root technique (SQRT). GLS was also used for the interaction model among 6- to 9-year-old children at baseline.

All models were compared with their null model to evaluate whether they were parsimonious and significant. The F estimators were significant for the main models (*p*-values ≥0.001 and ≤0.05).

### 3.2. Results at Baseline

Next, we tested whether there was an inverse relationship between PA levels and the total SDQ score. At baseline (Table 4), the result showed that indeed there was a difference between the levels of PA with regard to the total SDQ scores. Children with a high level of PA had less general mental health difficulties than children with a medium level of PA (Es = −0.11, t = −2.64, *p* = 0.01, Beta = 0.14) and a low level of PA (Es = −0.20, t = −4.26, *p* = 0.001, Beta = 0.25) among 4- and 5-year-old children. There was no significant difference between medium and low level of PA in this age range.

Among children between 6 and 9 years of age, a higher PA level was significantly associated with lower scores of total difficulties (Table 4). In all of these comparisons, the effect sizes were small to medium [72]. Only in study participants aged 6–9 years, there was a large effect size for the difference between high- and low-level activity (Es = −0.33, t = −8.28, *p* = 0.001, Beta = 0.35).

Compared to children with a low level of PA, the mean score of total difficulties at baseline was lower in children of 10–17 years of age with a high level of PA (Es = −0.16, t = −6.11, *p* = 0.001, Beta = 0.18) and children with a medium level of PA (Es = −0.21, t = −8.75, *p* = 0.001, Beta = 0.24) (Table 4). In this age group, there was an unexpected result for the comparison between a medium and high level of PA (Table 4). The 10- to 17-year-old children with a high level of PA had significantly greater difficulties than children with a medium level of PA (Es = 0.05, t = 1.97, *p* = 0.05, Beta = 0.06).

The results for the second aim (negative association between SDQ-H/I symptoms and level of PA) at baseline are also reported in Table 4. Among preschool children (aged 4 and 5 years), a high level of PA was linked to a reduced SDQ-H/I score in comparison to a low level (Es = −0.41, t = −3.13, *p* = 0.01, Beta = 0.19) and a medium level of PA (Es = −0.28, t = −2.37, *p* = 0.05, Beta = 0.13), while no significant difference between low and medium levels of PA was observed. These results were similar among 6- to 9-year-old children (Table 4).

Among adolescents aged 10–17 years, the mean score of ADHD symptoms was lower at a medium level of PA than at a low level of PA (Es = −0.26, t = −4.56, *p* = 0.001, Beta = 0.12). Similar to the result for total difficulties, children with a high level of PA showed more symptoms of ADHD in comparison to children with a medium level (Es = 0.24, t = 3.85, *p* = 0.001, Beta = 0.11).

An objective of this study was to investigate the role of receiving an ADHD diagnosis in the relationship between PA and total difficulties. The interaction between levels of PA and diagnosis of ADHD (ADHD vs. controls) was not significant across all age groups. The results from the groups aged 6–9 and 10–17 years were χ^2^(2) = 1.10, *p* = 0.57, and χ^2^(2) = 3.20, *p* = 0.20, respectively. At the age group of 4–5 years, the sample size was not large enough to analyze the interaction between PA and both groups.

### 3.3. Results at Wave 1

The results for participants at wave 1 are reported in Table 4. Among children aged 6–9 years, the mean of total SDQ score was significantly lower at a high level of PA than at a low (Es = −0.21, t = −3.24, *p* = 0.01, Beta = 0.25) or medium level of PA (Es = −0.09, t = −2.28, *p* = 0.05, Beta = 0.11). There was no difference between low and medium levels in this age range. Adolescents aged 10–17 years showed a significant inverse and medium difference between a high and a medium level of PA in comparison to a low level of PA. However, the difference between a high and a medium level of PA was non-significant.

Although the mean score of SDQ-H/I was lower at a higher level of PA, the difference was only significant between a low and a high level of PA (Es = −0.12, t = −2.08, *p* = 0.05) with a small effect size (Beta = 0.16) and a medium and a high level of PA (Es = −0.08, t = −2.18, *p* = 0.05, Beta = 0.11) among 6–9-year-old children (Table 4). In the age group of 10–17 years, hyperactivity/inattention symptoms were lower for a medium level of PA than for a low level of PA (Es = −0.21, t = −3.27, *p* = 0.01, Beta = 0.10). The difference was not significant between a low and a high level. Adolescents aged 10–17 years showed more ADHD symptoms at a high level of PA in comparison to a medium level (Es = 0.26, t = 3.86, *p* = 0.001, Beta = 0.13).

In the children and adolescents aged between 10 and 17 years, there was no significant interaction (χ^2^(2) = 0.50, *p* = 0.77) between frequency levels of PA and the two groups of subjects with ADHD diagnosis and controls. At 6–9 years of age, the sample size was not large enough to conduct an interaction analysis.

### 3.4. Results at Wave 2

At wave 2, data were only available for adolescents aged 10–17 years (Table 4). The results showed that the mean total SDQ score at a low level of PA was higher than at a medium (Es = −0.26, t = −6.88, *p* = 0.001, Beta = 0.27) and a high level (Es = −0.20, t = −4.40, *p* = 0.001, Beta = 0.21).

Adolescents who were active at a medium level showed fewer ADHD symptoms (Es = −0.23, t = −3.46, *p* = 0.001, Beta = 0.12) than those with a low activity, but there was no significant difference between a low and a high level of PA with respect to SDQ-H/I score. The results in Table 4 show that adolescents and young adults with a high level of PA had more ADHD symptoms than those who were at a medium level of PA (Es = −0.22, t = 3.05, *p* = 0.01, Beta = 0.11), albeit the effect size was very small. The interaction was not significant at this time point (χ^2^ (2) = 1.77, *p* = 0.41).

## 4. Discussion

Since there is mixed empirical evidence with respect to the effect of PA on mental health in children and adolescents, this study aimed to further investigate this issue, expecting a negative relationship. General and specific (here ADHD as movement-related problem) developmental psychopathologies were chosen as probable differentiating factors.

The main finding of this large-scale, nationwide, epidemiologic, multi-cross-sectional study conducted in German children and adolescents of different developmental age groups at three different time points demonstrated that higher physical activity was associated with better general mental health and lower specific symptoms of hyperactivity and inattention. In addition, no interaction was observed between PA and the two ADHD diagnosis groups (ADHD vs. non-ADHD controls) regarding general psychopathological difficulties in children and adolescents. From these data, it can be concluded that the relationship between PA and mental health problems is not influenced by the diagnosis of ADHD; i.e., inverse relationships between PA and mental health problems were found similarly in subjects both with ADHD diagnosis and control children.

### 4.1. Physical Activity and General Developmental Psychopathology

Results from this study suggest that children and adolescents who were more physically active showed better general mental health and thus fewer mental health problems, assuming that a high level of PA may be a protective factor against psychological problems in children and adolescents. The results were similar in all tested age groups at baseline, wave 1, and wave 2, respectively; the effect size was between low (low-high comparison in the age group of 4–5 years at baseline, Beta = 0.11) and medium (low-medium comparison in the 10–17 age group at wave 2, Beta = 0.27). These findings from the KiGGS study are consistent with previous work indicating a positive relationship between PA and mental health. In their systematic review, Dale et al. concluded that PA may lead to positive mental health among 5- to 17-year-old children and adolescents [19]. In the study by Vella et al., sport participation was a significant predictor of mental health trajectories in young children between the age of 4 and 12 years. In fact, children who did not participate in sport were 2.1 (odds ratio, OR) times more likely to be part of the high difficulty trajectory, representing the highest risk of mental health problems, than those who participated in sport [23]. Anxiety and depression symptoms were higher (OR 1.43 and 1.88, i.e., moderate to severe compared to no symptoms) among 14- and 15-year-old, physically inactive children [18]. Internalizing (hazard ratio [HR] = 0.81) and externalizing (HR = 0.65 and HR = 0.71 for 1–3 times and ≥4 times per week, respectively) disorders had lower occurrence among 10- and 11-year-old children who were physically active three times or more a week compared with those who were never physically active [24]. The results of the current study are compatible with the conclusion of a systematic review and meta-analysis conducted by Rodriguez-Ayllon et al., who found that PA leaves a significant positive influence on mental health in children and adolescents [38].

Generally, in the present study, a low level of PA was related to a higher score of mental problems. The findings among preschool children aged 4–5 years and 6–9 year-old elementary-school children at baseline and wave 1 were not congruent with data from the above-mentioned meta-analysis by Rodriguez-Ayllon and colleagues. The authors did not find a positive relationship between PA and mental health in younger children [38]. This difference might refer to the diverse methodology and (mostly small) sample sizes reported in the papers they included in their study. Likewise, O’Brien et al. reported no significant relationship between PA and mental health disorders in early school-aged Australian children [35]. The sample size in our study was quite large, and the positive relationship between PA and mental health was found at two time points in 6- to 9-year-old children. The effect sizes for the comparisons between low and high levels of PA were moderate to high (Beta = 0.25 to 0.35) in children aged 4–5 and 6–9 years at baseline and wave 1, but the effect sizes with respect to other comparisons between frequency levels of PA were quite low. These findings may imply that only at high levels can PA provide a buffer against mental problems in preschool and elementary school children.

Interestingly, in 10- to 17-year-old children at all three time points, adolescents with a high level of PA had more mental problems than those with a medium level of PA. However, this result was only weakly significant at baseline. Even though it opposes most of the previous works [18,24,38], this finding is highly consistent with results from a study by Hartman and co-workers [37]. These authors demonstrated that children, who were more physically active showed a somewhat higher risk of behavioral problems (SDQ measure), although their study was conducted among younger children. The result might be, at least partly, explained by the fact that some adolescents take part more often in activities and sports that are more risky and not always recreational. Moreover, parents’ expectations considering specific kinds of activities as useful and appropriate for their youngsters might have played a role. In the present study, the type of PA was not indicated.

### 4.2. Physical Activity and ADHD Symptoms

Our results showed that symptoms of ADHD were associated with a low level of PA. At baseline, parents reported significantly fewer symptoms of hyperactivity/inattention for children who were at the medium or high levels of PA compared to those who were at a low level. The gradual decrease in the SDQ-H/I score from the low to the high PA class suggests that PA may be considered as a health-related behavioral factor for ADHD symptoms at medium and high levels of PA, although the effect sizes were low to medium (0.13 to 0.21). These findings were consistent with several types of research that showed benefits of PA in children with ADHD [47,48,59]. Since the difference between levels of PA was not significant through age groups, it might be concluded that there is a specific range of PA that may serve as a protective element against hyperactivity/inattention symptoms.

Among preschool (4–5 years of age) and elementary-school children (6–9 years) at baseline and wave 1, the high level of PA was significantly associated with fewer reported symptoms of ADHD. However, the effect sizes were again small. Children who were at the medium level of PA had fewer symptoms of ADHD in comparison to low-level active children, but this association was not significant among 4 to 5- and 6 to 9-year-old children, respectively. This finding is inconsistent with previous research that reported that more PA was associated with more hyperactivity/inattention symptoms [36,37]. This disagreement may originate from the different methods for measuring PA. Kostyrka-Allchorne et al. used a parent-rated questionnaire (e.g., swimming, football, dancing) [36], whereas Hartman et al. used accelerometry (ActiGraph GT3X), which has some limitations for separating ADHD-related hyper-motoric behaviors from other physical activities [37].

Our data demonstrated that in children between 10 and 17 years, the score of ADHD symptoms was significantly higher at high level of PA as compared to the medium level at all three time points. The level of PA among adolescents may be a key factor in determining mental health and ADHD symptoms by the parents. It should be considered that effect sizes for these results were small, although they were statistically significant.

The findings of this part of the study showed that there is no similar and repetitive pattern for the association between PA and SDQ-H/I. In addition, associations were generally weak and a firm conclusion cannot be drawn regarding the effect of PA on ADHD symptoms. 

### 4.3. Physical Activity and Dose-Response Effects

As already mentioned above, children with a low level of PA had more mental problems than those who presented with a medium or high level. These results were significant and constant in most comparisons at the three measurement points over time. It seems that the intensity and frequency of PA are two important factors in order to produce a positive effect on mental health. Moreover, other studies have reported that a higher level of PA is associated with better mental health [1,3,8,24,25,46,47,48]. For example, children who were physically active 1–3 or ≥4 times per week had a lower rate of internalizing and externalizing disorders in comparison to those who were never active [24]. McMahon et al. grouped PA into four categories from least active (0–3 days) to sufficiently active (14 days) within two weeks and found that a higher frequency of PA was associated with better well-being and lower levels of anxiety and depression in adolescents [25]. In another study it has been reported that the more frequent and intense exercise was associated with better self-regulation, emotional ability, and decreased hyperactivity and impulsivity in children [8]. Gallego-Méndez et al. reported that as the frequency of PA increased, ADHD children showed better scores in health-related quality of life [3]. In a systematic review of exercise intervention in children and adolescents with ADHD, it was demonstrated that at least parents or teachers reported considerable positive effects of exercise on ADHD in studies evaluating the long-term effects of exercise [46]. Hence, it may be concluded that higher levels of PA might be associated with better mental health.

It should be considered that the range of effect sizes regarding the association between the frequency level of PA and its positive effect on mental health was always low to moderate. This holds not only for the current study but also for the several systematic reviews and meta-analyses that reported either weak effects or methodological shortcomings in this relationship [38,48,52]. Nevertheless, PA might be a supplementary protective factor for mental health.

This study has several strengths, which could overcome some weaknesses of earlier studies on the issue. The sample size was large and adequate for complex statistical analyses at the three sequential time points. Furthermore, three different developmental age groups could be separately tested at the three time points, representing roughly independent samples, but showing similar results for general and specific psychopathology (here: ADHD) at these three time points. Hence, this study presents reliable results, clarifying some of the uncertainties of research carried out thus far. However, this study also faced some limitations, for example, using only parents’ reports for detecting psychopathology, transforming data in some of the models, using a one-item question for evaluating PA, and not specifying the type of PA. In addition, our findings are not representative of the German population due to oversampling of study participants from eastern parts of Germany, and no weighting factor was included in our analyses. Further research on PA and mental health problems should keep equally large sample sizes, like ours, and a developmental perspective (if possible in a longitudinal design). Moreover, multi-informant questionnaires for child behavior and PA, combined with actigraphy, could improve the demanded replication and extension of this practically important area of research.

## 5. Conclusions

The present post hoc study suggests that a medium and/or high level of PA is associated with both good general mental health and low ADHD symptoms in children and adolescents from the community. Thus, PA did not differentiate between its effects on general versus specific aspects of psychopathology. These findings support the WHO recommendation on the importance of regular PA among children and adolescents [73]. However, since our findings are associative, they should not be interpreted in a causal way. Only longitudinal investigations of the topic may be able to decide whether unfavorable general mental health may be an obstacle for a systematic application of PA, or whether PA may be considered as a supplementary factor in preventing and treating the psychopathology of children and adolescents.

## Figures and Tables

**Table 1:** Descriptive characteristics of the study population at baseline.

Variables/Age Group	4–5 Years (4.49 ± 0.05)		6–9 Years (7.52 ± 1.11)		10–17 Years (13.37 ± 2.26)	
	*N*	Mean ± SD	*N*	Mean ± SD	*N*	Mean ± SD
Participants	1935		4136		7830	
SDQ						
Total difficulties	1904	8.31 ± 4.49	4059	8.43 ± 5.25	7564 ^†^	8.18 ± 5.26
Hyperactivity/inattention	1906	3.40 ± 2.20	4062	3.34 ± 2.36	7571 ^†^	2.7 ± 2.8
PA	1829		3867		7614	
Low	566 (30.9%)		819 (21.2%)		3208 (42.1%)	
Medium	702 (36.3%)		1377 (35.6%)		2385 (31.3%)	
High	561 (30.7%)		1671 (43.2%)		2021 (26.5%)	
ADHD	58		332		860	
Diagnosed	29 (50%)		166 (50%)		430 (50%)	
Control	29 (50%)		166 (50%)		430 (50%)	
**Descriptive characteristics were used in the model after control of covariates**						
	**SDQ-total** ***N* (Mean)**	**SDQ-H/I** ***N* (Mean)**	**SDQ-total** ***N* (Mean)**	**SDQ-H/I** ***N* (Mean)**	**SDQ-total** ***N* (Mean)**	**SDQ-H/I** ***N* (Mean)**
PA						
Low	548 (9.38 ± 4.86)	549 (3.79 ± 2.31)	791 (9.90 ± 5.60)	797 (3.78 ± 2.37)	3040 (8.38 ± 5.2)	3045 (2.73 ± 2.11)
Medium	695 (8.18 ± 4.4)	695 (3.37 ± 2.17)	1362 (8.69 ± 5.32)	1366 (3.40 ± 2.40)	2287 (7.52 ± 4.96)	2284 (2.69 ± 2.10)
High	533 (7.41 ± 4.04)	553 (3.02 ± 2.09)	1648 (7.42 ± 4.77)	1652 (3.05 ± 2.26)	1932 (8.16 ± 5.17)	1936 (3.13 ± 2.22)
Total	1796 (8.31 ± 4.51)	1797 (3.39 ± 2.21)	3801 (8.39 ± 5.24)	3815 (3.33 ± 2.35)	7259 (8.05 ± 5.14)	7265 (2.83 ± 2.15)

^†^ Numbers after deleting outliers.

**Table 2:** Descriptive characteristics of the study population at wave 1.

Variables/Age Group	6–9 Years (7.55 ± 1.10)		10–17 Years (13.53 ± 2.26)	
	*N*	Mean ± SD	*N*	Mean ± SD
Participants	2548		5953	
SDQ				
Total difficulties	2534 ^†^	8.53 ± 4.92	5865 ^†^	7.90 ± 4.87
Hyperactivity/inattention	2546	3.29 ± 2.25	5861 ^†^	2.75 ± 2.08
PA	2535		5615	
Low	305 (12%)		1389 (24.7%)	
Medium	1120 (44.2%)		2985 (53.2%)	
High	1110 (43.8%)		1241 (22.1%)	
ADHD	158		646	
Diagnosed	79 (50%)		323 (50%)	
Control	79 (50%)		323 (50%)	
**Descriptive characteristics were used in the model after control of covariates**				
	**SDQ-total** ***N* (Mean)**	**SDQ-H/I** ***N* (Mean)**	**SDQ-total** ***N* (Mean)**	**SDQ-H/I** ***N* (Mean)**
PA				
Low	202 (9.74 ± 5.21)	203 (3.62 ± 2.23)	1145 (8.65 ± 4.95)	1369 (2.72 ± 2.07)
Medium	818 (8.58 ± 4.81)	824 (3.32 ± 2.24)	2452 (7.50 ± 4.60)	2926 (2.61 ± 2.01)
High	708 (8.25 ± 5.02)	713 (3.20 ± 2.28)	1031 (7.93 ± 5.09)	1224 (3.06 ± 2.20)
Total	1728 (8.58 ± 4.97)	1740 (3.31 ± 2.26)	4628 (7.88 ± 4.82)	5519 (2.74 ± 2.07)

^†^ Numbers after deleting outliers.

**Table 3:** Descriptive characteristics of the study population at wave 2.

Variables/Age Group	10–17 Years (14 ± 1.99)	
	*N*	Mean ± SD
Participants	4598	
SDQ		
Total difficulties	4502	7.13 ± 4.89
Hyperactivity/inattention	4472 ^†^	2.51 ± 1.97
PA	4367	
Low	1214 (27.8%)	
Medium	2173 (49.8%)	
High	980 (22.4%)	
ADHD	494	
Diagnosed	247 (50%)	
Non-diagnosed	247 (50%)	
**Descriptive characteristics were used in the model after control of covariates**		
	**SDQ-total** ***N* (Mean)**	**SDQ-H/I** ***N* (Mean)**
PA		
Low	936 (7.84 ± 5.04)	1185 (2.47 ± 1.95)
Medium	1737 (6.59 ± 4.62)	2119 (2.35 ± 1.92)
High	768 (7.26 ± 5.19)	953 (2.78 ± 2.06)
Total	3441 (7.08 ± 4.89)	4257 (2.48 ± 1.97)

^†^ Numbers after deleting outliers.

**Table 4:** Results at baseline, wave 1, and wave 2 showing associations of physical activity (PA), mental health problem (Strengths and Difficulties Questionnaire [SDQ] total), and ADHD symptoms (SDQ hyperactivity/inattention subscale (SDQ-H/I)).

	Age Groups	PA	SDQ-Total			SDQ-H/I		
			Estimate (SE)	T Value	Coefficient (std.)	Estimate (SE)	T Value	Coefficient (std.)
Baseline	4–5 years	Low-medium	−0.09 (0.05)	−1.944	−0.11	−0.12 (0.12)	−1.01	−0.06
		Low-high	−0.20 (0.05)	−4.26 ***	−0.25	−0.41 (0.13)	−3.13 **	−0.19
		Medium-high	−0.11 (0.04)	−2.64 **	−0.14	−0.28 (0.12)	−2.37 *	−0.13
	6–9 years	Low-medium	−0.106(0.04)	−2.60 **	−0.11	−0.13 (0.10)	−1.31	−0.06
		Low-high	−0.33 (0.04)	−8.28 ***	−0.35	−0.48 (0.09)	−4.84 ***	−0.21
		Medium-high	−0.23 (0.03)	−6.85 ***	−0.24	−0.35 (0.08)	−4.25 ***	−0.15
	10–17 years	Low-medium	−0.21 (0.02)	−8.75 ***	−0.24	−0.26 (0.05)	−4.56 ***	−0.12
		Low-high	−0.16 (0.02)	−6.11 ***	−0.18	−0.02 (0.06)	−0.30	−0.01
		Medium-high	0.05 (0.02)	1.97 *	0.06	0.24 (0.06)	3.85 ***	0.11
Wave 1	6–9 years	Low-medium	−0.11 (0.06)	−1.79	−0.14	−0.04 (0.05)	−0.69	−0.05
		Low-high	−0.21 (0.06)	−3.24 **	−0.25	−0.12 (0.06)	−2.08 *	−0.16
		Medium-high	−0.09 (0.04)	−2.28 *	−0.11	−0.08 (0.03)	−2.18 *	−0.11
	10–17 years	Low-medium	−0.22 (0.03)	−7.18 ***	−0.25	−0.21 (0.06)	−3.27 **	−0.10
		Low-high	−0.21 (0.03)	−5.58 ***	−0.24	0.04 (0.08)	0.59	0.02
		Medium-high	0.01 (0.03)	0.40	0.01	0.26 (0.06)	3.86 ***	0.13
Wave 2	10–17 years	Low-medium	−0.26 (0.03)	−6.88 ***	−0.27	−0.23 (0.06)	−3.46 ***	−0.12
		Low-high	−0.20 (0.04)	−4.40 ***	−0.21	−0.01 (0.08)	−0.16	−0.01
		Medium-high	0.057 (0.04)	1.40	0.06	0.22 (0.07)	3.05 **	0.11

* Significant at 0.05 level, ** significant at 0.01 level, *** significant at 0.001 level, coefficient (std) = Beta, PA: physical activity, SDQ-total: total Strengths and Difficulties Questionnaire score, SDQ–H/I: symptoms of ADHD measured using the score of the hyperactivity/inattention subscale of SDQ.

## Data Availability

We received the raw data from the Robert Koch Institute. The extracted data backing up the findings of the current analyses are accessible from the first author (P.G.) on request.
